# Hydrogel-mediated delivery of platelet-derived exosomes: Innovations in tissue engineering

**DOI:** 10.1016/j.heliyon.2024.e24584

**Published:** 2024-01-17

**Authors:** Mostafa Saberian, Niloofar Abak

**Affiliations:** a Department of Medical Laboratory Sciences, School of Allied Medical Sciences, Tehran University of Medical Sciences, Tehran, Iran; b Hematology and Transfusion Science Department, School of Allied Medical Sciences, Tehran University of Medical Sciences, Tehran, Iran

**Keywords:** Platelet-derived exosomes, Hydrogel, Drug delivery, Tissue regeneration, Tissue engineering

## Abstract

In this scholarly review, we conduct a thorough examination of the significant role played by platelet-derived exosomes (Plt-Exos) and hydrogels in the fields of tissue engineering and regenerative medicine. Our detailed investigation highlights the central involvement of Plt-Exos in various physiological and pathological processes, underscoring their potential contributions to diverse areas such as wound healing, neural rejuvenation, and cancer progression. Despite the promising therapeutic aspects, the notable variability in the isolation and characterization of pEVs underscores the need for a more rigorous and standardized methodology. Shifting our focus to hydrogels, they have emerged as promising biomaterials relevant to tissue engineering and regenerative medicine. Their unique characteristics, especially their chemical and physical adaptability, along with the modifiability of their biochemical properties, make hydrogels a captivating subject. These exceptional features open avenues for numerous tissue engineering applications, facilitating the delivery of essential growth factors, cytokines, and microRNAs. This analysis explores the innovative integration of Plt-Exos with hydrogels, presenting a novel paradigm in tissue engineering. Through the incorporation of Plt-Exos into hydrogels, there exists an opportunity to enhance tissue regeneration endeavors by combining the bioactive features of Plt-Exos with the restorative capabilities of hydrogel frameworks. In conclusion, the cooperative interaction between platelet-derived exosomes and hydrogels indicates a promising path in tissue engineering and regenerative medicine. Nevertheless, the successful execution of this approach requires a deep understanding of molecular dynamics, coupled with a dedication to refining isolation techniques.

## 1 Introduction

Platelet-derived extracellular vesicles (pEVs) have emerged as significant mediators in a range of physiological and pathological processes. Their involvement has been demonstrated in various areas, including regenerative medicine, wound healing, neural regeneration, cancer progression, atherosclerosis, liver fibrosis, retinal microvasculature, thrombosis, inflammation, autoimmune diseases, and interactions between neutrophils and endothelial cells [[1], [2], [3], [4], [5], [6], [7], [8], [9], [10], [11], [12], [13], [14], [15], [16]].

In the realm of regenerative medicine, platelet-derived extracellular vesicles (pEVs) have demonstrated potential for therapeutic applications. Their capacity to induce cellular differentiation and enhance musculoskeletal or neural regeneration has been explored (1). However, the acquisition and characterization of pEVs exhibit wide heterogeneity, deviating from the recommendations of the International Society for Extracellular Vesicles (1). Further research is necessary to standardize isolation methods and provide a more comprehensive characterization of pEVs. In the field of atherosclerosis research, pEVs have been implicated in modulating atherosclerotic pathogenesis. They play a role in intercellular communication between platelets and other blood and vascular cell types, such as endothelial cells (ECs), leukocytes, and vascular smooth muscle cells (VSMCs) [4].

Platelets serve as a crucial connection between atherosclerosis (AS), inflammation, and thrombosis. Platelet-derived extracellular vesicles (P-EVs), encompassing both microparticles (MPs) and exosomes (EXOs), actively participate in the pathogenesis of AS. They contribute to the progression of the disease through multiple mechanisms [4]. P-EVs have the capacity to interact with cancer cells, either promoting disease progression or harboring antitumor functions. The outcome is dependent on their specific binding partners within the tumor microenvironment [10].

P-EVs) also play a role in liver fibrosis. Autophagy in hepatic stellate cells (HSCs) inhibits the release of fibrogenic EVs when these cells are stimulated by platelet-derived growth factor (PDGF). This mechanism serves to attenuate liver fibrosis [14]. Autophagy in hepatic stellate cells (HSCs) mitigates liver fibrosis by suppressing the release of fibrogenic EVs [14]. In the retinal microvasculature, interactions between pericytes and endothelial cells are pivotal for maintaining the integrity and functionality of the neurovascular unit. Platelet-derived exosomes (PL-EXOs) play a role in intercellular communication and contribute to the pathological progression of diseases such as retinopathy of prematurity, retinal vein occlusion, and diabetic retinopathy.

PL-EXOs can serve as biomarkers for these diseases [7]. Platelet activation serves as a triggering factor for inflammation and atherosclerosis. During thrombosis, activated platelets release numerous extracellular vesicles that not only promote coagulation but also interact with inflammatory cells [15]. Platelet-derived microparticles (PMPs) drive disease progression through various mechanisms. These include binding to both cancer cells and non-transformed cells in the tumor microenvironment, transferring platelet-derived contents to target cells, exerting promoting or modulating effects on cellular recipients based on their cargo content, contributing to cancer comorbidities through roles in coagulation and thrombosis, and interacting with inflammatory cells [8]. The potential of platelet-derived extracellular vesicles (pEVs) to enhance neutrophil-endothelial cell interactions has been a subject of investigation. P-EVs efficiently bind to neutrophils or endothelial cells (EC), resulting in the upregulation of activation markers on these cells. They can facilitate transient attachments or stable adhesion between flowing neutrophils and deposited vesicles or EC through diverse molecular mechanisms, including CD62P and integrin [2].

p-EVs encompass numerous cytokines, growth factors, and miRNAs, each playing functional roles in tissue reconstitution. To ensure the efficient transfer of *p*-EVs to injured tissues and prevent their destruction and unintended spread, delivery through structures such as hydrogels is essential. P-EVs, known for their regenerative properties, complement the characteristics of hydrogels, which are insoluble in water, permeable, porous, and biodegradable. The synergistic combination of these features presents an exceptional approach for tissue regeneration and wound healing.

To affirm the confirmatory role of *p*-EVs in tissue repair and initiate their clinical use, there arises a need for frameworks facilitating the effective delivery of *p*-EVs to damaged organs. Recently, various types of hydrogels have been formulated and continue to be expanded. Their non-toxic and biocompatible characteristics render them valuable tools for drug delivery, including the delivery of *p*-EVs. This review aims to compile information and present a comprehensive overview of this field.

## 2 Search strategy

A systematic search was performed in databases, specifically PubMed and Web of Science, utilizing the following terms: (((((hydrogel) AND (tissue regeneration)) OR (hydrogel)) AND (tissue engineering)) AND ((((exosome) OR (Extracellular Vesicle)) OR (Cell-Derived Micro particles)) OR (Cell-Derived nanoparticles))) OR (Platelet-Derived Exosome) with a filter for the last 5 years. Articles were subsequently screened based on title, abstract, and full-text relevance. Additional articles were obtained from the reference lists of key studies. (Table 1).

**Table 1:** Search strategy and article screening.

Step	Description	Number/Detail
Database Selection		PubMed, Web of Science
Key Concepts Identified		- Exosome - Extracellular Vesicle - Cell-Derived Microparticles - Cell-Derived nanoparticles - Platelet-derived exosome - hydrogel - tissue regeneration - tissue engineering
Search Query	(((((hydrogel) AND (tissue regeneration)) OR (hydrogel)) AND (tissue engineering)) AND ((((exosome) OR (Extracellular Vesicle)) OR (Cell-Derived Microparticles)) OR (Cell-Derived nanoparticles))) OR (Platelet-Derived Exosome) Filters: in the last 5 years	
Filters Applied		- Last 5 years (2018–2023 September) - English language
Initial Articles Retrieved	Inclusions covered pertinent topics according to keywords	284
Title/Abstract/Full-texts Screening	The most consistent with the objectives of the study	82 with reasons
Articles Excluded	articles involving synthetic vesicles, non-hydrogel polymer scaffolds, chemical drug treatments, nursing methods in wound healing, and those exceeding a five-year publication date unrelated to the study's purpose.	87 excluded
Total Articles in Review		196

The data collection and processing model adhered to the principles inherent in crafting narrative review articles. A comprehensive search was conducted utilizing specified keywords and related terms. Inclusion criteria encompassed articles published within the past five years, along with a limited select number of articles from prior years that addressed fundamental topics pertinent to the research. Conversely, exclusion criteria were rigorously applied, excluding articles falling within the following categories.

1. Articles designed for the synthetic vesicles, such as liposomes, for drug delivery in tissue repair.
2. Articles employing non-hydrogel polymer scaffolds in the context of tissue repair.
3. Articles primarily concentrating on wound models and treatment using commercially available chemical drugs.
4. Articles centered on care and nursing methodologies in the context of wound healing.
5. Articles surpassing the five-year publication timeframe that lacked relevance to the study's objectives.

## 3 Historical context about extracellular vesicles and hydrogels

Wolf first identified the ‘lipid-rich particles' released from platelets as platelet-derived extracellular vesicles (P-EVs), and Warren et al. provided a comprehensive representation of the release of P-EVs from activated platelets. However, due to a lack of understanding of EVs, in-depth research was limited for many years. With advancements in research and a better understanding of EVs, more knowledge has been conserved, and advanced research procedures are now available to support further study into EVs (83).

Around 1900, the term ‘hydrogel' first appeared in scientific literature, referring to a colloidal gel of inorganic salts. In the 1960s, Wichterle and Lim introduced the concept of hydrogels as water-swollen crosslinked macromolecular networks. They notably utilized poly (2-hydroxyethyl methacrylate) (pHEMA) gels for soft contact lenses. By the 1970s, PEG hydrogels cross-linked via gamma radiation were developed. In 1993, PEG-PLA hydrogel cross-linked through photopolymerization was introduced, and in 1998, hydrogels from self-assembling artificial proteins emerged. The year 2000 witnessed the development of stereocomplex dextran hydrogel, and in the 2010s, smart hydrogels were introduced (84).

With the advancement of tissue engineering and biomaterials, researchers have recognized that combining EVs with biomaterials can address the limitations of EVs in specific applications for tissue reconstitution. Hydrogels, as traditional and classical biomaterials, have played a crucial role in tissue reconstruction. However, hydrogels inherently lack bioactivity. The utilization of hydrogels loaded with EVs serves to enhance EV stability and facilitates their transfer to the defect site for sustained in situ release. Numerous studies have demonstrated that EV-loaded hydrogels exert significant effects on tissue regeneration. (85).

## 4 Therapeutic effects of platelet-derived exosomes in tissue regeneration

Platelet-derived exosomes have surfaced as a promising therapeutic avenue in the realms of tissue engineering and regenerative medicine [17]. These exosomes are derived from platelets and contain various bioactive molecules such as growth factors, cytokines, and microRNAs [1]. Research has demonstrated their ability to stimulate cellular differentiation, facilitate musculoskeletal or neural regeneration, and promote wound healing [1,14,18,19]. Platelet-derived exosomes are obtained from various sources, including whole blood, plasma, platelets, and platelet-rich plasma (PRP) and resulting in notable variabilities in their composition and functions. Exosomes released from platelets of individuals with different diseases often carry pathogenic factors, serving as biomarkers for disease detection (86). The extraction protocol of P-EVs can be categorized into four steps: 1-isolation of platelet-rich plasma (PRP) from whole blood, 2-more isolation of platelets from PRP, 3- platelet stimulation to induce the release of EVs and 4- EVs extraction through the process of differential centrifugation. (83).

In addition to differential centrifugation methods, the isolation and purification of platelet-derived exosomes (PLT-Exos) can be achieved through conventional extraction techniques such as filter centrifugation, density gradient centrifugation, immune magnetic bead procedures, phosphatidylserine affinity methods, and chromatography. according to recent assessments, the evaluation of exosome-specific markers like CD9, CD63, and CD81 can be conducted using flow sorting. Additionally, direct observation through electron projection fluorescence microscopy can be employed for computational analysis (87).

The protein contents of PRP-Exos and their intercellular connections may be influenced by different platelet activation mechanisms. In the activation of platelet-rich plasma (PRP), the use of single calcium gluconate was found to be less potent than that of thrombin or a combination of thrombin and calcium gluconate. Additionally, some exosomes activated by calcium displayed rough surfaces in transmission electron microscopy (TEM) images. Furthermore, the concentration of PRP-Exos triggered by thrombin was observed to be lower compared to that induced by calcium gluconate or the combination of thrombin and calcium gluconate. Exosomes released from platelets can be stimulated by various agonists; however, the efficiency of activation and the cytokine profiles vary. Notably, the use of a combination of thrombin and calcium gluconate for platelet-rich plasma (PRP) activation has a more pronounced effect compared to thrombin or calcium alone, impacting both the quality and quantity of exosomes (88).

Future studies and advancements in the isolation and purification of platelet-derived exosomes (PLT-Exos) should aim to minimize the impact of experimental variables and enhance the sensitivity of detection markers for clinical disorders. Combining micro-magnetic resonance and fluorescence immunoassay techniques can synergistically improve the yield of purification, thereby enhancing the sensitivity and accuracy of platelet exosome extraction (87).

### 4.1 Therapeutic consequences of platelet-derived exosomes in diabetic wound healing

One study investigated the utilization of pEVs in the context of chronic diabetic wound healing and skin regeneration [18]. In their investigation, the researchers encapsulated pEVs within a hydrogel and applied it topically to diabetic wounds in a rat model. The results indicated that the combination of pEVs with the hydrogel notably accelerated the rate of wound closure. Furthermore, there was an observed increase in the expression of markers associated with wound healing, enhanced regeneration of granulation tissue, and an upregulation of the expression of growth factors involved in angiogenesis [18]. These findings suggest a potential therapeutic effect of the pEV-loaded hydrogel in promoting diabetic wound healing and tissue regeneration.

### 4.2 Treatment outcomes with PRP

Another study was centered on the isolation and characterization of platelet-derived exosomes obtained from human platelet-rich plasma (PRP) [20]. In their research, the investigators employed standard methods to isolate exosomes from PRP and subsequently characterized them using multiple techniques, including Western blot analysis, transmission electron microscopy, and array analysis. The findings indicated that the exosomes derived from PRP exhibited a molecular signature consistent with platelet-derived exosomes. Moreover, the morphological features observed were in line with the typical characteristics of exosomes. Moreover, they demonstrated that PRP exosomes exhibited lower levels of most analytes in comparison to non-activated platelets. However, they found relatively higher levels of analytes that have been consistently validated as components of the protein cargo of platelet exosomes [20]. This study provides valuable insights into the molecular composition of platelet-derived exosomes and their potential therapeutic applications.

several studies have explored the potential application of platelet-derived exosomes in hair growth and regeneration. a narrative review synthesized information on the most promising regenerative treatments for alopecia, encompassing mesenchymal stem cell implantation, treatment with mesenchymal stem cell-derived secretome, and therapies involving platelet-rich plasma derived from blood [1]. The authors discussed the evidence supporting these treatments and highlighted opportunities for improving efficacy and innovative strategies for promoting clinical application [1].

Another study evaluated the therapeutic options for premature ovarian insufficiency (POI) and discussed the potential use of stem cell and exosome therapies in POI treatment [21]. The authors of this study reviewed diverse regenerative treatment modalities, one of which is platelet-rich plasma intra-ovarian infusion, that are being developed to address the limited treatment options for POI patients. They emphasized the need for precise design components to accelerate the conversion of these emerging therapies into clinical treatments [21].

Another study investigated the effect of platelet lysate (PL) exosomes on ameliorating glucocorticoid-induced osteoporosis. ‌ Prolonged and improper use of glucocorticoids (GCs) is known to often lead to a reduction in bone mineral density and the development of osteonecrosis. PL is described as the second generation of PRP. It is considered a cost-effective alternative to commercially recombinant growth factors (GFs) with lower immunogenicity, hence exhibiting an extensive application landscape. In addition to GFs both platelet lysate (PL) and platelet lysate-derived exosomes (PL-Exos) serve as valuable carriers of microRNA, and a crucial cluster that regulates bone homeostasis. PL enhances osteogenesis and angiogenesis in vivo and as a result, accelerates bone repair. The information presented indicates that PL-derived exosomes sustained the osteogenic differentiation of MSC and the vascularization of EPC more proficiently than PL alone. the collected data presents that both PL and PRP have substantial anti-apoptotic and pro-proliferation effects (89).

Sydney et al. showed evidence-based results on the use of platelet exosomes for skin rejuvenation. Top-quartile responders experienced a complete effect during the early induction phase (4–6 weeks), and participants indicated normalized skin health by global skin health analysis. Sub-cohort analysis in the skin health induction phase exhibits remarkable refinement in erythema, melanin pigmentation, color evenness, and wrinkles due to hyaluronic acid and collagen production as a result of using PRP at 6 weeks. this is the first description of a topical platelet-derived exosome product on skin health. (90).

In contrast to the studies mentioned earlier, the work by Yang Jiao and colleagues evaluated the effect of platelet-derived exosomes in the neutrophil extracellular trap (NET) formation and organ laceration. Platelets are powerful activators of NET formation. This activation occurs through direct interactions between platelets and neutrophils, facilitated by cell adhesion molecules or soluble mediators such as high mobility group box 1 (HMGB1) and chemokines released by platelets. The findings from their study indicate that exosomal HMGB1 and/or miRNAs from platelets, contribute to an excessive NET formation in septic shock and leading to acute lung injury. the study suggests that Platelet consumption could impede NET formation and ameliorate lung injury in various murine models of lung injury (91).

This information shows controversies with other mentioned studies. However, more research exhibits the restorative effects of platelet-derived exosomes in multiple damaged organs.

## 5 The cellular and molecular impacts of platelet-derived exosomes on tissue restoration

Platelet-derived exosomes have garnered interest as promising therapeutic entities for tissue repair, owing to their capacity to release growth factors and cytokines that facilitate tissue regeneration [14]. Saumell-Esnaola et al. (2022) isolated and characterized platelet-derived exosomes from human PRP. They found that activated platelets release a greater quantity of exosomes compared to non-activated platelets. and these exosomes exhibited a molecular signature consistent with platelet-derived exosomes. Furthermore, array analysis showed that these exosomes had lower levels of most analytes compared to non-activated platelet-derived exosomes but higher levels of validated components of the protein cargo [14]. This study provides valuable insights into the molecular composition of platelet-derived exosomes and validates a method for isolating highly pure platelet exosomes.

TGF-β is a potent growth factor involved in wound healing. Shi et al. (2021) investigated the effects of TGF-β loaded exosomes on ischemic wound healing, both in vitro and in vivo. They observed that TGF-β-loaded exosomes stimulated cell proliferation, migration, tube formation, and the development of skin organoids in vitro. In a rabbit model of ischemic wounds, topical application of a fibrin sealant containing TGF-β-loaded exosomes facilitated complete healing, including the reacquisition of hair follicles and sebaceous glands [22]. This study demonstrates the potential of TGF-β loaded exosomes for enhancing wound healing.

In a study by Guo et al. (2017), the impact of PRP-derived exosomes on chronic cutaneous wounds was investigated using a diabetic rat model. The findings revealed the effective induction of endothelial cell and fibroblast proliferation and migration by PRP-derived exosomes, leading to enhanced angiogenesis and re-epithelization in chronic wounds. additionally, the study identified the involvement of the YAP pathway in mediating these effects [23]. This is the first and key study in the field of PRP-derived exosomes loaded in alginate hydrogel for injury repair and provides evidence for the role of PRP-derived exosomes in promoting wound healing. Miller et al. (2022) investigated the regenerative effects of platelet-derived purified exosomes on human endometrial cells. The results indicated that treatment with platelet-derived exosomes led to increased cell proliferation and enhanced wound-healing capacity in various types of endometrial cells [24]. This study highlights the potential therapeutic application of platelet-derived exosomes in promoting endometrial regeneration. In their 2023 review, Feng et al. explored the potential therapeutic effects of exosomes in diabetic wounds. Exosomes derived from various cellular sources were demonstrated to enhance cell proliferation, migration, angiogenesis, nerve repair, vascular renewal, and wound vascularization. the ability to deliver different therapeutic loads renders exosomes a promising target for precision therapeutics in diabetic wounds [25].

In Rodeo's (2023) overview of orthobiologics, including platelet-rich plasma as a potential treatment for soft tissue healing deficiencies, current data suggests that orthobiologics are more symptom-modifying than structure-modifying. However, an enhanced understanding of underlying mechanisms may pave the way for more targeted treatments [26]. Tamir et al. (2016) utilized cryo-electron tomography to study the structural organization of platelet-derived microparticles (PMPs). PMPs were found to contain functional cell adhesion machinery including integrins and cytoskeletal systems. Interestingly, Glanzmann thrombasthenia patients with deficient αIIbβ3 integrin exhibited altered cytoskeletal organization in their PMPs compared to healthy controls [27]. This study provides insights into the native molecular organization of PMPs.

Henriques-Antunes et al. (2019) investigated the kinetics of small extracellular vesicle (SEV) delivery and its influence on skin tissue regeneration. They found that multiple applications of SEVs yielded superior regenerative effects compared to a single dose, Moreover, both diabetic and non-diabetic wounds treated with SEV-loaded hydrogel exhibited enhanced wound closure [28]. This study underscores the importance of considering the kinetics of SEV delivery for optimal tissue regeneration. Zhou et al. (2020) isolated exosomes from tick saliva and salivary glands, revealing their role in inhibiting wound healing through downregulation of CXCL12 and upregulation of IL-8. Inhibition of CXCL12 or IL-8 further delayed exosome-mediated cell migration and wound healing, indicating a protective role for these chemokines at the tick bite site [29]. This study provides insights into how tick salivary exosomes delay wound healing to facilitate successful blood feeding.

Zhang et al. (2023) investigated the effects of PDGFBB on menstrual blood-derived stromal cells (MenSCs) and their exosomes in alleviating endometrial fibrosis. PDGFBB not only improved the biological functions of MenSCs, including viability, migration, and stemness but also enhanced the anti-fibrotic properties of MenSC-derived exosomes [30]. This study implies that PDGFBB can enhance the therapeutic potential of MenSC-derived exosomes for endometrial fibrosis. Wang et al. (2021) identified low-dose radiation-induced circ-METRN as a crucial regulatory mechanism in glioblastoma progression and radioresistance acting through miR-4709–3p/GRB14/PDGFRα pathway. Exosomal circ-METRN derived from glioblastoma cells treated with low-dose radiation promoted tumor progression and radioresistance by influencing recipient cell cytokine secretion profiles [31]. This study emphasizes the significance of exosomal circ-METRN in glioblastoma progression and radioresistance.

Xuchang Liu et al. evaluated the alleviating effect of exosomes derived from platelet-rich plasma (PRP) on knee osteoarthritis. They induced an osteoarthritis (OA) model in vitro using IL-1β and observed a substantial reduction in the proliferation and migration of chondrocytes. additionally, they noted an apparent increase in the apoptosis of chondrocytes. PRP-exosomes yielded more favorable outcomes in enhancing chondrocyte proliferation and migration while diminishing apoptosis compared to PRP alone. A possible mechanism that plays a crucial role in the anti-inflammatory effects mediated by PRP exosomes is the Wnt/β-catenin signaling pathway. WNT protein activates the canonical Wnt signaling cascade through a paracrine approach, influencing cell proliferation, cell fate determination, and differentiation in cellular homeostasis. The accumulation of β-catenin and Wnt5a in IL-1β-induced osteoarthritis chondrocytes elevated the expression of downstream transcription factors, such as RUNX2. However, few studies have demonstrated the regulatory role of PRP in the Wnt/β-catenin signaling pathway. The detailed exploration of this signaling pathway in PRP-Exos-mediated chondrocyte production has not yet been fully recognized (92).

**Fig. 1:**
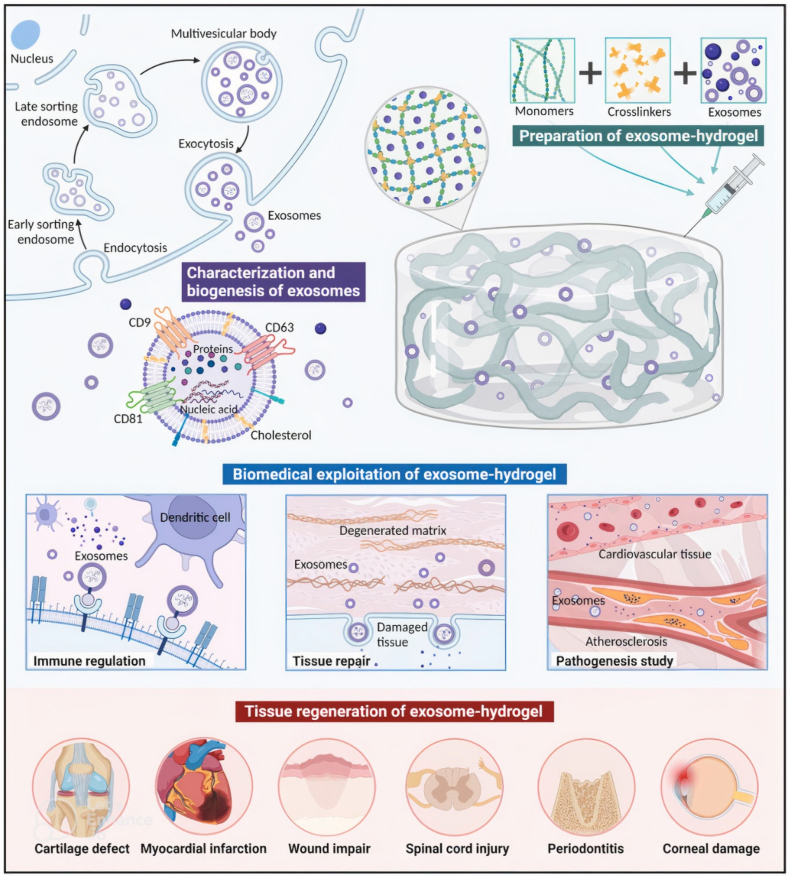
Schematic view of exosomes loading in hydrogel and their tissue regenerative effects [32].

Ming Tan et al. investigated how thrombin-induced platelet-derived exosomes can reduce the expression of PDGFR-β in vascular smooth muscle cells (SMCs). The increase in levels of miR-223, miR-339, and miR-21 in thrombin-activated platelet-derived exosomes, and their subsequent delivery to vascular SMCs, resulted in a decrease in PDGF-β expression in vitro. This, in turn, led to the inhibition of SMC proliferation and an elevation in SMC apoptosis. The regulatory mechanism in this pathway may occur via miR-223, as increased levels of miR-223 in platelet-derived exosomes were observed. However, additional suppression of PDGFR-β may also depend on another miRNA and mRNA-regulatory complex, such as Ago 2. The MAPK signaling pathway mediates multiple cell proliferation responses, and PDGF considerably triggers ERK2 in a Ras-dependent manner (93). A schematic view from exosome loading in hydrogel, biomedical utilization and their applications in tissue repair has been shown in Fig. 1.

## 6 The application of hydrogels in tissue engineering

Hydrogels have emerged as promising biomaterials for tissue engineering and regenerative medicine applications [33]. These materials consist of crosslinked polymer chains forming three-dimensional network structures that closely resemble living tissues, due to their high water content, soft structure, and porosity [34]. Hydrogels can be derived from various materials, including natural polymers such as collagen, alginate, and chitosan [33,35,36], as well as synthetic polymers like polyaniline and polypyrrole [37]. Synthesis of these hydrogels can be achieved through chemical or physical methods, providing control over physical parameters such as size, elastic modulus, swelling, and degradation rate [33].

### 6.1 Composite hydrogels

A notable area of interest in hydrogel research involves the development of composite hydrogels [38]. Incorporating conducting polymers such as polyaniline and polythiophene into hydrogels has been pursued to create conductive biomaterials with applications in tissue engineering [37]. These conductive polymers exhibit promising conductivity as bioactive scaffolds for tissue regeneration and allow cells or tissues cultured on them to be stimulated by electrical signals. However, their mechanical brittleness and poor processability limit their application. To overcome these limitations, researchers have developed conductive polymeric composites. These composites are based on conductive polymers and biocompatible biodegradable polymers [37].

### 6.2 Decellularized extracellular matrix biomaterials

Decellularized extracellular matrix (dECM) is another type of biomaterial that has gained attention in regenerative medicine. It retains crucial biomolecules essential for tissue maintenance and repair [39]. It has been employed in engineering various types of tissues or organs, utilizing different scaffold types like injectable hydrogels, electrospun scaffolds, and bioprinted scaffolds [39]. The preservation of natural molecules in dECM offers advantages over synthetic or naturally derived hydrogels, promoting cell growth, function, differentiation, angiogenesis, and other biological processes [33,39].

### 6.3 Mesenchymal stem cells in hydrogels for regenerative medicine

Mesenchymal stem cells (MSCs) have also been integrated into hydrogels for regenerative medicine applications. MSC-based nanoparticles and scaffolds show potential in wound healing, bone and cartilage engineering, cardiac disorders, and neurological disorders [40]. Nanotherapies can encapsulate MSCs within hydrogels or load them with drugs for targeted delivery [40]. Additionally, nanofibers from polymer scaffolds such as polycaprolactone (PCL), poly-lactic-*co*-glycolic acid (PLGA), silk fibroin, collagen, chitosan, alginate, hyaluronic acid (HA), and cellulose have been used to directly support or grow MSCs [40].

### 6.4 Polyelectrolyte hydrogels

Polyelectrolyte hydrogels have also shown promise in tissue engineering and regenerative medicine applications. These emerging materials possess tunable biochemical properties that closely resemble the extracellular matrix in mammalian bodies [41]. Due to their ionic nature, they exhibit electrical conductivity and biocompatibility. Polyelectrolyte hydrogels carrying anionic, cationic, and ampholytic moieties have been developed for various tissue engineering applications including drug delivery systems and anti-biofouling coatings [41]. Alginate, a naturally occurring anionic polymer derived from brown seaweed, has garnered attention in tissue engineering applications. Alginate-based biomaterials, including gels, sponges, films, and fibers, have been developed for tissue repair and regeneration due to their biocompatibility, low toxicity, cost-effectiveness, and mild gelation properties [42]. Alginate can undergo chemical modifications to acquire additional functional properties and has been utilized as a component in drug delivery applications [42].

### 6.5 Gradient stiffness hydrogels

Gradient stiffness hydrogels have also been explored in tissue engineering, designed to exhibit varying stiffness across their structure, mimicking the natural gradients found in tissues [43]. These hydrogels have demonstrated effects on cell differentiation, proliferation, migration, and apoptosis. Gradient stiffness hydrogels provide greater control over cellular behavior and structural stability compared to conventional hydrogels with uniform stiffness [43].

### 6.6 Silk fibroin

Silk fibroin, another biomaterial gaining attention in tissue engineering applications, can be easily reconstituted into different forms such as films, mats, hydrogels, and sponges through various fabrication techniques [36]. Silk fibroin scaffolds exhibit excellent mechanical properties biodegradability biocompatibility bio-absorbability making them suitable for bone cartilage teeth mandible tissue skeletal muscle vascular tissue regeneration [36].

### 6.7 Stimuli-responsive hydrogels

Stimuli-responsive supramolecular hydrogels are smart materials that undergo sol-gel or gel-sol transition in response to changes in their surroundings. These hydrogels can respond to various external stimuli, including mechanics, thermal energy, light, electric fields, ultrasonics, pH, enzyme levels, and more [44]. Stimuli-responsive supramolecular hydrogels find applications in tissue engineering, drug delivery, wound healing, imaging, and medical diagnostics, due to their ability to modulate the microenvironment at the site of interest [44].

### 6.8 Nanocomposite hydrogels

In regenerative medicine applications, nanocomposite hydrogels have been explored. These composite materials combine nanomaterials with 3D hydrogel networks, enhancing their properties and functions through physical or chemical means [45]. Nanocomposite hydrogels offer multi-functionality by incorporating nanoparticles with distinct physical-chemical properties into the hydrogel matrix. While they show potential in various biomedical applications, they face challenges related to material design, synthesis, processing, characterization, regulatory approval, and safety concerns, among others [45].

## 7 Loading of platelet-derived exosomes in hydrogel structure

The strategy of loading platelet-derived exosomes into hydrogels has been investigated to enhance their therapeutic effects. In a study conducted by Yang et al. (2020) [18], the combination of human umbilical cord MSC-derived exosomes (hUCMSC-exos) with Pluronic F-127 hydrogel for diabetic wound healing was explored. The results indicated accelerated wound closure, increased expression of CD31 and Ki67 (markers of angiogenesis and cell proliferation), enhanced regeneration of granulation tissue, and upregulated expression of vascular endothelial growth factor (VEGF) and transforming growth factor beta-1 (TGFβ-1). This study provides evidence for the efficacy of hUCMSC-exos loaded in hydrogel for promoting diabetic wound healing.

Another study by Zhang et al. (2022) [46] investigated the use of platelet-rich plasma-derived exosomes (PRP-Exos) incorporated in thermosensitive hydrogel for cartilage protection in subtalar osteoarthritis. They found that PRP-Exo released from the hydrogel promoted cell proliferation, migration, and chondrogenic differentiation, while inhibiting inflammation-induced chondrocyte degeneration in vitro. In vivo experiments showed that PRP-Exo incorporated gel increased local retention of exosomes, inhibited apoptosis and hypertrophy of chondrocytes, enhanced their proliferation, and potentially delayed the development of subtalar osteoarthritis. This study demonstrates the potential therapeutic effect of PRP-Exo loaded in a hydrogel system for cartilage protection.

A review article by Nallakumarasamy et al. (2022) [47] summarized the advanced applications of mesenchymal stromal cell-derived extracellular vesicles, including exosomes, in wound healing. They discussed the use of various types of exosomes, including platelet-rich plasma-derived exosomes and engineered exosomes, in hydrogels to prolong their contact time at the recipient site within the target tissue. The review highlighted that these biomaterial-based approaches hold potential for managing chronic wounds by influencing intercellular signaling, immunomodulation, neoangiogenesis, collagen synthesis, and neoepithelization.

A study by Lu et al. (2023) [48], investigated the incorporation of platelet-derived exosomes loaded with recombinant Yap1 into GelMA hydrogel to promote the rejuvenation of tendon stem/progenitor cells (TSPCs) for functional tendon regeneration. The treatment with this functionalized hydrogel increased TSPCs' stemness and differentiation potentials in vitro and facilitated tendon regeneration in an Achilles tendon defect model in vivo. This study underscores the potential of platelet-derived exosome-loaded hydrogels for promoting functional tendon regeneration.

Xu et al. (2018) [49] investigated the use of a chitosan/silk hydrogel sponge loaded with platelet-rich plasma exosomes (PRP-Exos) for wound healing in a diabetic rat model. The study found that PRP-Exos, either alone or in combination with Curcuma zedoaria polysaccharide (ZWP), led to wound contraction and increased epidermal thickness compared to control groups. However, the combination therapy of PRP-Exos/ZWP exhibited superior wound-closing efficacy compared to individual treatments, attributed to upregulated collagen synthesis and deposition, as well as enhanced angiogenesis at the wound site. A review of all mentioned studies about exosome loaded hydrogels for tissue reconstitution available in Table 2.

**Table 2:** An overview of the exosomes loaded hydrogels studies.

Year	Type of hydrogel	Source of exosomes	Target tissue
Guo sc et al. (2017)	Alginate	human umbilical cord MSC-derived exosomes PRP-exosomes	Chronic cutaneous wound (in rat model)
Xu et al. (2018)	chitosan/silk hydrogel	platelet-rich plasma exosomes	wound healing in a diabetic rat model
Henriques-Antunes et al. (2019)	a Light-Triggerable Hydrogel	SEVs isolated from human umbilical cord blood mononuclear cells	skin
Yang et al. (2020)	Pluronic F-127 hydrogel	human umbilical cord MSC-derived exosomes	diabetic wound
Cheng et al. (2021)	sprayable hydrogel	cerium oxide nanoparticles (CeONs) and an antimicrobial peptide (AMP)	wound
Shi A et al. (2021)	fibrin sealant (TISSEEL)	Platelet exosomes	wound
Guo et al. (2021)	Decellularized tooth matrix	human dental pulp stem cell derived exosomes	tooth
Mu et al. (2022)	peptide PPFLMLLKGSTR-modified hyaluronic acid hydrogel	hypoxia-stimulated mesenchymal stem cell-derived exosome	spinal cord injury
Fan L et al. (2022)	electroconductive hydrogels	bone marrow stem cell-derived exosomes	spinal cord injury
Zhang et al. (2022)	Poloxamer-407 and 188 mixture-based thermoresponsive hydrogel	platelet-rich plasma-derived exosomes (PRP-Exo)	Cartilage
Lu et al. (2023)	GelMA hydrogel	platelet-derived exosomes loaded with recombinant Yap1	Achilles tendon (defect model)
Li Q et al. (2023)	silk fibroin patches	miR146a-loaded engineered exosomes	tooth

## 8 Methods for loading exosomes in hydrogels

EVs are nanoscale or microscale vesicles secreted by cells, playing important biological roles [50]. Studies have shown that extracellular vesicles derived from mesenchymal stem cells promote tissue repair and regeneration [50]. However, due to their rapid clearance capacity, EVs are unable to persistently act at specific sites for tissue repair (49).

Hydrogels, known for their good biocompatibility and porous properties, serve as carriers for EVs, prolonging their retention at specific sites and slowing down their release [50]. Several strategies have been developed for loading EVs into hydrogels, including encapsulation within the hydrogel matrix or surface modification of the hydrogel with EVs.

One study investigated the use of extracellular vesicle-loaded hydrogels for tissue repair and regeneration [50]. The researchers reviewed different types of hydrogels, discussing factors influencing their capacity to carry and release EVs. They also summarized several strategies for loading EVs into hydrogels and characterizing the resulting EV-loaded hydrogels. Furthermore, the study delved into application strategies for these EV-loaded hydrogels in tissue regeneration and repair [50].

Another study focused on exosomes derived from nicotine-stimulated macrophages accelerating atherosclerosis through miR-21–3p/PTEN-mediated vascular smooth muscle cell migration and proliferation [51]. In vivo studies using mice fed a high-fat diet and treated with nicotine subcutaneously were conducted. They observed increased plaque exosome retention in vivo after nicotine administration. In vitro experiments showed that isolated exosomes from nicotine-treated macrophages promoted vascular smooth muscle cell migration and proliferation. They also identified miR-21–3p as a key miRNA enriched in these exosomes, directly targeting PTEN to enhance their effects [51].

A review article summarized the advanced applications of stimuli-responsive drug delivery systems for inflammatory arthritis treatment [19]. They discussed various internal and external stimuli that can be utilized to trigger drug release from nanomaterial-based carriers. They explored the progress and challenges with the use of stimuli-responsive nanomaterials to manage inflammatory arthritis, considering pathological changes such as cartilage degeneration, synovitis, and subchondral bone destruction [19].

Another study investigated the use of electroconductive hydrogels loaded with bone marrow stem cell-derived exosomes for spinal cord injury repair [52]. They found that these exosome-loaded hydrogels modulated microglial polarization and enhanced neuronal and oligodendrocyte differentiation of neural stem cells. The combination of electroconductive hydrogels and exosomes significantly decreased inflammation, enhanced local neural stem cell recruitment, and promoted neuronal regeneration in an animal model of spinal cord injury [52].

One study focused on the combination of umbilical cord-derived mesenchymal stem cell-derived exosomes with Pluronic F127 hydrogel for chronic diabetic wound healing and complete skin regeneration [18]. In vitro, experiments showed that this combination therapy accelerated wound closure rate, increased expression of CD31 (a marker for blood vessels) and Ki67 (a marker for cell proliferation), enhanced regeneration of granulation tissue, upregulated expression of vascular endothelial growth factor (VEGF) and transforming growth factor beta-1 (TGFβ-1). In vivo experiments using a diabetic rat model demonstrated improved wound healing compared to control treatments [18].

## 9 Implications applications

### 9.1 Regenerative medicine

Exosomes, extracellular vesicles with crucial roles in cell-cell communication, have demonstrated significant potential in regenerative medicine. These vesicles can be derived from various sources, including MSCs, which have been extensively studied for their regenerative properties [53]. MSC-derived exosomes have been investigated for their therapeutic potential in various applications, including tissue repair and regeneration. The International Society for Extracellular Vesicles (ISEV) has issued a position statement that provides guidelines on the minimal information required for studies of extracellular vesicles (MISEV2018) [54]. This document emphasizes the importance of reporting specific information beyond mere description to support claims about the function and activities of exosomes. Several review articles discuss the role and therapeutic potential of MSC-derived exosomes in various contexts such as osteoarthritis [55], wound healing [50], periodontal regeneration [56], bone tissue regeneration [40], skin regeneration [57], dental pulp regeneration [58]. These reviews provide comprehensive overviews of current knowledge on these topics.

Original research articles investigate different aspects related to exosome-based therapies. For instance, one study focuses on using engineered exosomes loaded with miR146a to promote diabetic wound healing by targeting IRAK1 [59]. Another study explores the use of silk fibroin patches loaded with miR146a-loaded engineered exosomes to revitalize avulsed teeth through tooth regeneration processes facilitated by an ontogenesis-related developmental microenvironment [58]. Overall, the evidence suggests that MSC-derived exosomes have significant potential in regenerative medicine. These exosomes can promote tissue repair and regeneration through various mechanisms such as paracrine signaling, modulation of inflammation, and enhancement of angiogenesis. Nevertheless, further research is needed to fully understand their therapeutic effects and optimize their delivery systems.

### 9.2 Hydrogels as drug delivery applications

Drug-loaded hydrogels have emerged as promising systems for controlled drug delivery in various biomedical applications. These hydrogels, characterized by crosslinked polymer chains and three-dimensional network structures, have the capability to absorb large amounts of fluid [44]. hydrogels closely resemble living tissues due to their high water content, soft structure, and porosity [44]. they can be classified into natural polymers (e.g., chitosan, alginate) and synthetic polymers (e.g., polyvinyl alcohol, polyethylene glycol) based on their composition [60].

The use of hydrogels for drug delivery offers several advantages such as sustained release kinetics, protection of drugs from degradation, and targeted delivery to specific sites within the body [61]. The choice of hydrogel material depends on factors such as biocompatibility, mechanical properties, and desired drug release profile [62]. Natural polymers are often preferred due to their biodegradability and biocompatibility compared to synthetic polymers [60]. Numerous studies have focused on the development of drug-loaded hydrogels using different types of drugs including small molecules, proteins/peptides, nucleic acids, and nanoparticles. These drugs can be encapsulated within the hydrogel matrix or conjugated onto the polymer chains [63]. The release mechanism from hydrogel nanoparticles is complex and involves a combination of diffusion, matrix swelling, and chemical reactivity between the drug and matrix components [64].

#### 9.2.1 Solutions for drug-loaded hydrogel improvement

Various strategies have been employed to enhance the performance of drug-loaded hydrogels. For example, incorporating stimuli-responsive elements into hydrogel systems allows for triggered release in response to external stimuli such as temperature variations or pH changes additionally, hybrid hydrogels composed of multiple polymers or incorporating nanoparticles have been developed to improve mechanical properties, drug loading capacity, and release kinetics [65].

#### 9.2.2 Other roles of hydrogels

The application of drug-loaded hydrogels extends beyond traditional drug delivery to areas such as tissue engineering, wound healing, and regenerative medicine. Hydrogels can provide a 3D cell culture environment that mimics the native extracellular matrix and promotes cell proliferation and differentiation [44]. They can also be used as scaffolds for tissue regeneration by providing structural support and delivering bioactive molecules that promote tissue growth [66].

#### 9.2.3 Limitations of drug delivery with hydrogel

Despite the significant progress in the development of drug-loaded hydrogels, several challenges remain. The design of hydrogel systems should consider factors such as biocompatibility, stability, scalability, and ease of administration [61]. The choice of crosslinking method affects the mechanical properties and degradation rate of the hydrogel [62]. Furthermore, achieving precise control over drug release kinetics is crucial for therapeutic efficacy.

## 10 Targeted therapy by applying hydrogels

Targeted therapy using hydrogels has gained significant attention in the field of biomedicine, particularly in wound healing and tissue regeneration [67]. Several studies have explored the use of hydrogels for targeted therapy by incorporating various therapeutic agents.

### 10.1 Photoactive hydrogels for lesion repair

One study investigated multifunctional photoactive hydrogels (MPRHs) for wound healing acceleration [67]. MPRHs integrate light-based photothermal therapy (PTT) and photodynamic therapy (PDT) with the advantages of hydrogels [1]. The study discussed different types of MPRHs prepared using diverse photosensitizers and photothermal/photodynamic agents [67]. It also highlighted how PTT, PDT, and synergistic therapy can modulate bacterial microenvironments to inhibit infection.

### 10.2 Composite hydrogel therapeutic effects

Another study focused on a tumor microenvironment-adapted polypeptide hydrogel/nanogel composite for antitumor molecularly targeted inhibition and immunoactivation [68]. This composite was designed to achieve precise sequential drug release for enhanced molecularly targeted therapy and immune activation. In animal models, the composite effectively inhibited tumor growth by increasing tumor infiltration of CD8^+^ T cells and promoting macrophage polarization from M2 to M1 type [68].

### 10.3 Hydrogels as an RNA transfer system

Hydrogels have also been explored for RNA delivery in RNA-based therapeutics. One review provided an overview of the hydrogel loading of RNAs, highlighting their potential applications in controlled-release systems that minimize systemic toxicity while enhancing efficacy [69]. Hydrogels can exert precise spatiotemporal control over the release of RNA therapeutics, enhancing in vivo efficacy [69]. In the field of cancer diagnosis and therapy, molecularly imprinted polymer hydrogel nanoparticles have shown promise as synthetic antibodies [70]. These nanoparticles can enhance the binding and recognition of biomarkers overexpressed in cancer cells, enabling improved diagnosis and treatment [71].

### 10.4 Controlled release of exosomes from hydrogels

Extracellular vesicles have gained significant attention in recent years due to their potential therapeutic applications in various diseases, including cancer, cardiovascular disease, and tissue regeneration [54]. EVs are nanoscale or microscale vesicles secreted by cells that play important biological roles. They can be classified into different subtypes based on their biogenesis pathways and size range, including exosomes, microvesicles, and apoptotic bodies [54].

### 10.5 Extracellular vesicles as a carrier for drug delivery

One key area of research involving EVs is their use as drug-delivery vehicles. Several studies have investigated the loading of therapeutic agents into EVs for targeted delivery to specific tissues or cells. For example, Wang et al. developed a doxorubicin delivery platform using engineered natural membrane vesicle exosomes for targeted tumor therapy [72]. They loaded exosomes derived from immature dendritic cells with doxorubicin and demonstrated efficient targeting and drug delivery to αv integrin-positive breast cancer cells both in vitro and in vivo [73].

Similarly, Wu et al. loaded adipose-derived mesenchymal stem cells (ADMSCs) exosomes with VH298, a HIF-1α stabilizer, for targeted treatment of diabetic wounds [74]. They showed that intravenous injection of the ADMSC-exosome-loaded hydrogel effectively promoted wound healing by enhancing angiogenesis without overt toxicity [74]. In addition to delivering therapeutic agents directly to target cells or tissues, EVs themselves have been shown to possess regenerative properties.

Ju et al. reviewed the use of extracellular vesicle-loaded hydrogels for tissue repair and regeneration [50]. They discussed how mesenchymal stem cell (MSC)-derived EVs, especially those secreted by ADMSCs, can promote the repair and regeneration of various tissues. They highlighted the potential of hydrogels as carriers for EVs, allowing for prolonged retention in specific areas and controlled release of EVs [50].

### 10.6 EVs and immune system regulation

Another area of research involving EVs is their role in modulating immune responses. The use of MSC-derived exosomes as immunomodulatory agents has been investigated in several studies. For example, Zou et al. discussed the therapeutic potential and mechanisms of MSC-derived exosomes as bioactive materials in tendon-bone healing [75]. They reviewed studies showing that MSC-derived exosomes can promote angiogenesis and cell proliferation, reduce inflammation, and produce bioactive molecules involved in tissue repair [75].

## 11 Development of hydrogels as an advantage for loading platelet-derived exosomes

. In recent years, hydrogels have been developed for various applications including drug delivery, tissue engineering, wound healing, and cell transplantation [[76], [77], [78], [79]]. One specific area of interest is the development of hydrogels for loading platelet-derived exosomes. Platelet-derived exosomes have emerged as a promising therapeutic approach for tissue regeneration due to their ability to promote angiogenesis and modulate immune responses [80,81].

Hydrogels can serve as an ideal carrier system for delivering these exosomes to target tissues. Several studies have explored the advantages of hydrogel development for loading platelet-derived exosomes. Cheng et al. (2021) developed a sprayable hydrogel dressing loaded with cerium oxide nanoparticles (CeONs) and an antimicrobial peptide (AMP) for wound healing [76,82]. They demonstrated that the hydrogel dressing exhibited favorable reactive oxygen species-scavenging abilities and antimicrobial activities against representative bacterial strains. The study also showed that the hydrogel dressing had good biocompatibility when cultured with HaCaT cells in vitro.

Safari et al. (2022) investigated the characteristics of exosome-loaded hydrogels for skin regeneration [80]. They highlighted the advantages of using exosomes as a cell-free approach instead of the direct application of stem cells. The study discussed the design of an optimized delivery system using hydrogels for preserving exosome bioactivity and achieving controlled release during wound care.

Mu et al. (2022) developed a hypoxia-stimulated mesenchymal stem cell-derived exosome-loaded hydrogel for angiogenic treatment of spinal cord injury [78]. They demonstrated that the hydrogel exhibited thermal responsiveness and effectively delivered hypoxia-stimulated exosomes to the injured spinal cord, promoting angiogenesis and functional recovery. These studies provide evidence supporting the advantages of hydrogel development for loading platelet-derived exosomes in various applications, including wound healing, skin regeneration, and spinal cord injury treatment.

The use of hydrogels as carriers for exosomes allows for controlled release, preservation of bioactivity, and targeted delivery to specific tissues.

### 11.1 Limitations

Despite progress in the identification and isolation of EVs, questions about subpopulations of EVs remain unclear, and distinguishing exosomes from other small vesicles remains challenging. Additionally, the low concentration of EVs poses challenges for large-scale mass manufacture. Another issue is the storage conditions of EVs-loaded hydrogel; the storage of EVs to conserve their biological activity should be at −80 °C (94,95).

The stability, mechanical characteristics, and cell adhesion of natural hydrogels pose challenges and limit their efficacy for bone and cartilage regeneration. To overcome these limitations, covalent cross-linkers such as glutaraldehyde and *genipin*, as well as physical treatments like UV irradiation and heating, can be employed to enhance these features. On the other hand, the combination of synthetic polymers with inorganic materials and the utilization of scaffold materials can overcome these challenges. However, it's important to note that synthetic hydrogels, despite their advantages, may be limited by the absence of an indigenous tissue structure, which can restrict their usage (94,95).

The stability and maintenance of exosomes are a significant obstacle for clinical applications, as they are promptly destructed and rapidly removed via immune system responses upon in vivo injection. Achieving high therapeutic efficacy and specificity necessitates the optimization of administration procedures and the desirable delivery of exosomes. The transfer of exosomes to target cells, facilitated by the interaction between biomaterials and cellular behaviors, is an additional challenge that requires thorough examination (94,95).

### 11.2 Recent advances and future directions

Future studies should aim to clarify the genuine contribution of EVs to PRP. There is a crucial need to establish standardized conditions and confirm optimized protocols that define the obtaining process, dose, and origin of EVs. Moreover, assessing an integrated strategy that exploits the synergistic effects of combining PRP-derived fibrin and EVs can play a pivotal role in developing new applications. Nevertheless, these promising results establish a novel foundation for advancing research in the fields of regenerative medicine and drug delivery in the future (96). The development of preparation methods, polymer physics, material knowledge, and biomimetic properties has led to progress in hydrogels as biomaterials. However, numerous problems and clinical requirements still persist. Optimizing pore size tuning, degradability, and compatibility in standard forms can significantly enhance the maintenance and release profiles of exosomes in vivo. For instance, 3D printing has been employed to promote functional porosities, pore shapes, and geometries of hydrogel scaffolds. Additionally, the incorporation of materials like integrins into synthetic hydrogels can lead to tunable release and extended delivery (94).

Current developments aim to prolong the half-lives and enhance the purity of exosomes. Commonly, design strategies for composite gels involve combining diverse types of polymeric components to achieve unique features. Further studies are needed, especially regarding smart hydrogels like CRISPR gel. These gels can be altered to offer programmable features derived from conventional materials, thus providing spatiotemporally defined interactions with exosomes for clinical applications (94). Currently, multifunctional, smart, and microneedle (MN) patch hydrogels loaded EVs are important research topics and are being vigorously evaluated. Novel EV extraction technologies and specific sera and reagents are needed to confirm hydrogel-based therapeutic procedures. Breakthroughs in bioreactors, 3D cell culture, microfluidic-based hydrogel, microsphere preparation technology, microneedle patch fabrication, and hydrogel-based 3D bio-printing technology are expected to further advance EV-loaded hydrogel research (95).

## 12 Conclusion

This study has substantially broadened our understanding of the roles and potentialities of hydrogels in the realm of regenerative medicine and tissue reconstruction, with a special emphasis on exosome-infused hydrogels. Given their intrinsic characteristics such as pronounced water retention, biocompatibility, and adaptable mechanical attributes, hydrogels emerge as an optimal conduit for the delivery of platelet-sourced exosomes aimed at applications ranging from wound recovery to skin rejuvenation, and addressing conditions like retinopathy, inflammation, autoimmune disorders, and spinal cord interventions.

Notably, the deployment of hydrogels for exosome therapeutic delivery has garnered attention for its prospective capabilities in modulated release systems, potentially leading to augmented therapeutic efficacy while minimizing systemic side effects. Additionally, avant-garde hydrogel configurations, incorporating mesenchymal stem cell-derived nanoparticles and scaffoldings, stimulus-reactive adaptive hydrogels, and alginate-centric biomaterials, persistently demonstrate immense potential in the spheres of tissue engineering and regenerative therapeutics.

Despite the substantial advancements in this field, there still exist hurdles to the refinement of hydrogel configurations and the perfection of drug dissemination dynamics, meriting further scholarly investigation. This manuscript accentuates the pivotal contribution of hydrogels in the advancement of regenerative medicine and accentuates the imperative of continued research to truly capitalize on the capabilities of these phenomenal biomaterials.

## Ethics approval and consent to participate

The study was conducted following the highest ethical standards. The data presented in this manuscript are accurate and authentic.

## Funding

The authors received no financial support for the research, authorship, or publication of this article.

## Data availability statement

Data availability is not applicable to this article as no new data were created or analysed in this study.

## CRediT authorship contribution statement

**Mostafa Saberian:** Writing – review & editing, Writing – original draft, Validation, Supervision, Resources, Project administration, Investigation, Funding acquisition, Data curation, Conceptualization. **Niloofar Abak:** Writing – review & editing, Software, Resources, Investigation.

## Declaration of competing interest

The authors declare that they have no known competing financial interests or personal relationships that could have appeared to influence the work reported in this paper.
